# The Role of Lumen-Apposing Metal Stents in Transmural Endoscopic Drainage of Postinflammatory Pancreatic and Peripancreatic Fluid Collections

**DOI:** 10.1155/2021/4351151

**Published:** 2021-10-13

**Authors:** Mateusz Jagielski, Marek Jackowski

**Affiliations:** Department of General, Gastroenterological and Oncological Surgery, Collegium Medicum, Nicolaus Copernicus University, Torun, Poland

## Abstract

Rapid development of advanced gastrointestinal endoscopic techniques contributed to the appearance of new biomedical materials including polymers, which are used for the production of different types of endoprostheses. Endotherapy (ET) of postinflammatory pancreatic and peripancreatic fluid collections (PPFCs) with the use of lumen-apposing metal stent (LAMS) is an effective method of treatment. This paper describes the high efficacy of ET and its potential complications, which are mostly related to the design of the LAMS used. The high efficacy of LAMS in the transmural drainage of PPFCs is associated with lower safety of treatment. Complications of ET presented in the manuscript are mainly related to endoprosthesis' construction. This paper presents possible directions of development in the field of transmural LAMSs, which in the future may contribute to the invention of an innovative type of LAMS based on new biomedical technologies. Possibly, subsequent novel endoprosthesis projects, based on the above results, will be able to meet the current needs and requirements associated with endoscopic transmural drainage procedures in cases of postinflammatory PPFCs. The ultimate goal is to improve safety of minimally invasive techniques for treatment of the local consequences of pancreatitis.

## 1. Introduction

**Figure 1:**
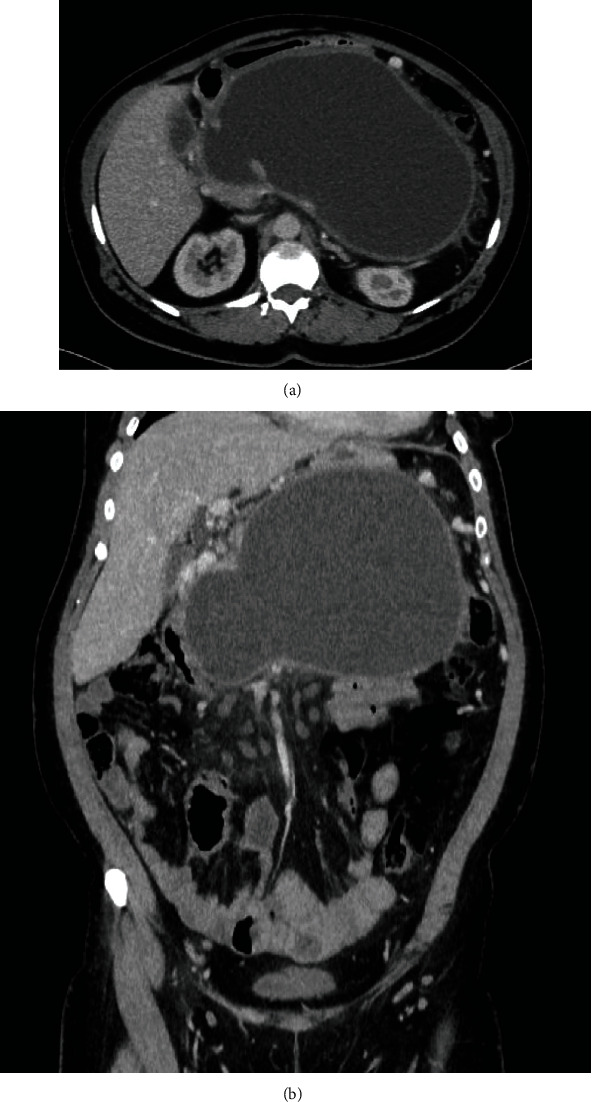
(a, b) A large pancreatic pseudocyst visualized by abdominal CECT in a female patient with acute pancreatitis.

**Figure 2:**
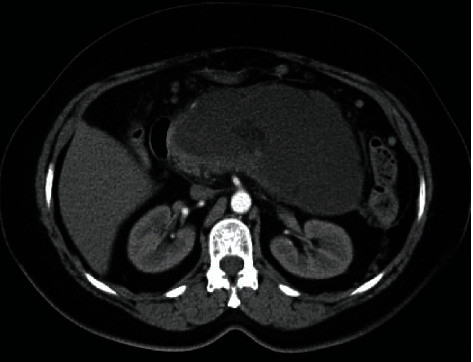
CECT of the abdomen in a patient with WOPN at week 8 of acute necrotizing pancreatitis.

Acute pancreatitis (AP) of moderate and severe clinical course is associated with high risk of local complications and organ failure leading to increased mortality [1–4]. Pancreatic and peripancreatic fluid collections (PPFCs) that may appear in the late phase of pancreatitis in the form of pancreatic pseudocysts (Figures 1(a) and 1(b)) and walled-off pancreatic necrosis (WOPN) (Figure 2). These types of PPFCs are the most common local complications of acute and chronic pancreatitis [1–7]. For many years, the traditional treatment of postinflammatory PPFCs in the late phase of pancreatitis relied on surgical methods [7–10]. However, there has been recent dynamic development of minimally invasive techniques, including endoscopic transluminal methods [7–12]. While endoscopic treatment is an established method of managing these complications, some aspects of endotherapy are still a source of much controversy [7, 13, 14]. One of the most debated issues in interventional endoscopy of local complications in pancreatitis is the use of transmural self-expanding metallic stents (SEMSs).

**Figure 3:**
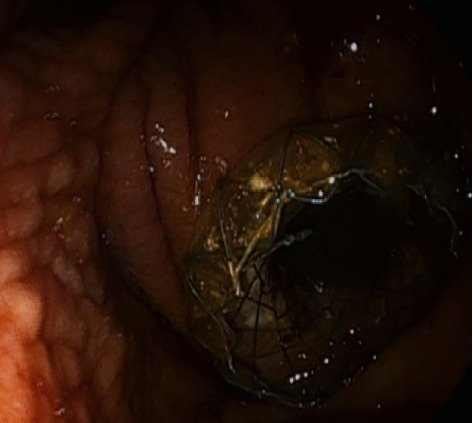
Passive transmural (transgastric) drainage of a postinflammatory pancreatic pseudocyst with a self-expanding stent.

**Figure 4:**
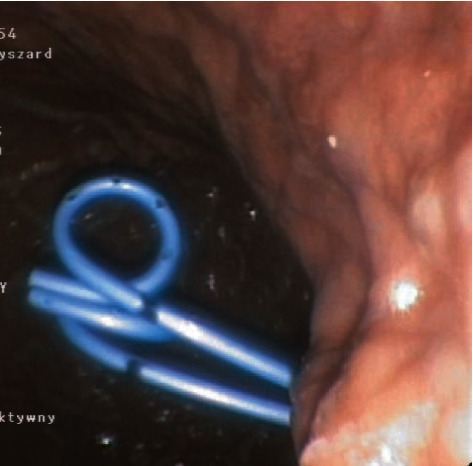
Passive transmural drainage of a pancreatic pseudocyst with two plastic double-pigtail stents.

**Figure 5:**
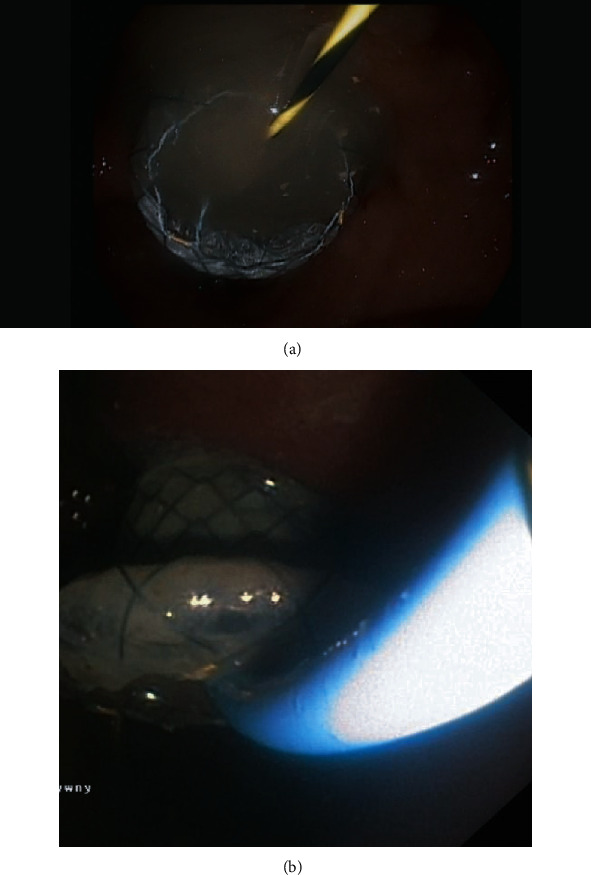
(a, b) Active transmural drainage of a WOPN. After the transmural fistula is created and a self-expanding stent (LAMS) is inserted transmurally through the fistula, (b) a nasal drain is introduced along (a) a guidewire into the necrotic area.

Endoscopic transmural drainage consists in creating a fistula between the lumen of the PPFC and gastrointestinal tract to allow for outflow of the content from the PPFC into the gastrointestinal tract [7, 13, 15, 16]. During an endoscopic ultrasound- (EUS-) guided procedure of endoscopic transmural drainage of postinflammatory PPFCs, this can be visualized in the endosonographic image through the wall of the upper gastrointestinal tract [7, 16, 17]. Afterwards, a transmural puncture of the PPFC is performed under EUS guidance with the use of a needle and widened with a cystostome to a diameter of 10 Fr using coagulation. This forms a transmural cystostomy, which joins the gastrointestinal tract and the lumen of the PPFC [7, 17]. The next step of the endoscopic procedure is mechanical (with a dilator) or pneumatic (with a high-pressure balloon) dilation of the pancreaticocystogastrostomy or pancreaticocystoduodenostomy [7, 16, 17]. Once dilated, a transmural SEMS (Figure 3) or plastic stent(s) (Figure 4) is introduced through the cystostomy to facilitate passive transmural drainage of the collection contents into the gastrointestinal tract [7, 17]. Passive transmural drainage (Figures 3 and 4) is an effective method of endoscopic treatment of pancreatic pseudocysts, whose contents are entirely liquid [7, 15, 16]. In case of necrotic PPFCs that contain both liquefied necrotic material and tissue fragments, it is necessary to use active transmural drainage, which consists in inserting an additional nasal drain through the transmural cystostomy to enable flushing of the collection cavity in the postoperative period (Figures 5(a) and 5(b)) [7, 17].

**Figure 6:**
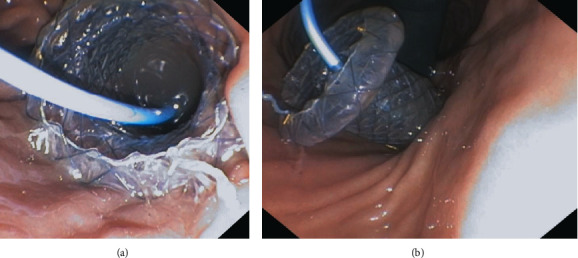
(a, b) Active transmural drainage. Transmurally/transgastrically placed LAMS in a patient undergoing endoscopic drainage of a WOPN.

Development of advanced endoscopic techniques has led to rapid advancements in biomedical materials, including polymers for manufacturing endotherapeutic devices. Currently, there is a wide variety of transmural endoprostheses of different sizes, shapes, and designs for endoscopic treatment of postinflammatory PPFCs [7]. These endoprostheses are divided into two groups. The first group includes plastic stents, usually made of teflon or polyethylene [7, 17]. The second group includes SEMSs, often referred to as “lumen-apposing metal stents” (LAMSs) that are used in the treatment of postinflammatory pancreatic local complications [7]. For many years, the only type of endoprosthesis available for use in transmural drainage was plastic double-pigtail stents [17, 18]. However, LAMSs (Figures 6(a) and 6(b)) have been attracting increasing interest as a relatively new option in endoscopy [19–23]. LAMSs are a special type of SEMS used in a variety of gastrointestinal endoscopic procedures. They are made of nitinol wire and are fully covered with a silicone membrane [19–23].

However, the role of LAMSs in transmural drainage remains unclear [7, 17–23]. This paper describes the outcomes of LAMS-based endoscopic treatment of postinflammatory PPFCs. Building on the authors' own experiences, this paper addresses the technical and structural features of transmural SEMSs and their usability in real-world clinical practice. The authors discuss the selection criteria for an appropriate type of endoprosthesis for transmural drainage of local complications of pancreatitis. Technical parameters of transmural endoprostheses are discussed in detail, with particular attention to endoscopic treatment complications associated with stent design. A number of novel methods have been presented for treating complications of endoscopic transmural drainage with the use of LAMSs. The main purpose of this study was to clarify the role of LAMSs in the transmural drainage of postinflammatory PPFCs.

In addition, the authors present an endoscopist's input regarding an ideal transmural endoprosthesis to improve the outcomes of endotherapy in postinflammatory PPFCs.

## 2. Materials and Methods

Prospective analysis of treatment outcomes in patients with postinflammatory PPFCs in late phase (>4 weeks) of pancreatitis, who received endoscopic treatment at the Department of General, Gastroenterological, and Oncological Surgery, Ludwik Rydygier Collegium Medicum, in Bydgoszcz, Nicolaus Copernicus University, in Toruń from 2018 to 2021.

The study was approved by the Ethics Committee at the Collegium Medicum of the Nicolaus Copernicus University and was conducted in accordance with the Declaration of Helsinki. All patients provided informed consent for endoscopic procedures.

The diagnosis of pancreatitis, the criteria of clinical and morphological categorization, and all the definitions of local and systemic complications were based on the 2012 revised Atlanta classification [1–4]. The standards for conservative treatment for pancreatitis were based on international guidelines [24, 25]. Conservative treatment relied primarily on dietary treatment with intensive intravenous fluid therapy and analgesia. Moreover, additional treatment methods were used depending on concomitant organ impairment and the patient's overall clinical condition. Each individual case of pancreatitis (medical records and imaging results) was thoroughly discussed during interdisciplinary meetings of senior staff. Decisions were made regarding further management of the patient and the potential rationale for interventional treatment.

### 2.1. Study Inclusion Criteria

All patients with clinical symptoms of PPFCs due to acute or chronic pancreatitis were enrolled. The patients underwent endoscopic drainage procedures. Qualification for endoscopic treatment was based on the clinical picture and imaging results, primarily abdominal contrast-enhanced computed tomography (CECT). The start of endoscopic treatment was postponed until the collection became encysted at the latest. If it was necrotic, the necrotic material collected within the cavity became liquified and a WOPN was formed, which occurred four weeks from the onset of the disease and was determined on the basis of imaging examinations of the abdominal cavity.

### 2.2. Study Exclusion Criteria

Patients with PPFCs that were not a consequence of pancreatic inflammatory disease were excluded from the study. The study also excluded patients with postinflammatory PPFCs without clinical symptoms and those who had undergone surgery in the pancreatic region. Patients who had undergone interventional treatment in the early phase (<4 weeks) of AP were also excluded.

### 2.3. Selection of the Type of Endoscopic Management [7, 26]

In patients with symptomatic PPFCs in the late phase of pancreatitis, transmural drainage using the single transluminal gateway technique (SGT) was performed if endoscopic ultrasound revealed that the distance between the wall of the collection and the gastrointestinal wall did not exceed 30 mm. In patients with sterile pancreatic pseudocysts, the method of choice was passive transmural drainage. In patients with infected pancreatic pseudocysts or WOPN (both sterile and infected), the method of intervention was active transmural drainage.

In the event that drainage with the single transluminal gateway technique (SGT) was ineffective and the fluid collection spreads beyond the lesser sac, multiple transluminal gateway technique (MTGT) was used. This technique has also been used in cases of multilocular postinflammatory PPFCs. If the necrotic areas were infected or transmural drainage was unsuccessful for WOPN patients, direct endoscopic necrosectomy was performed.

If endoscopic techniques with transmural access were ineffective, additional access to the collection cavity was created using percutaneous drainage (transperitoneal or retroperitoneal) or transpapillary drainage (through the major duodenal papilla). Endoscopic retrograde pancreatography (ERP) revealed communication between the main pancreatic duct (MPD) and the PPFC cavity.

### 2.4. Endoscopic Procedures

Endoscopic procedures were performed under general anesthesia with tracheal intubation. All patients provided informed consent for this procedure. All were performed by a single endoscopist, and the procedure entailed carbon dioxide insufflation and use of a linear echoendoscope (Pentax EG3870UTK, Pentax Medical, Tokyo, Japan), duodenoscope (Olympus TJF-Q180V, Olympus Corporation, Tokyo, Japan), and gastroscope (Olympus GIF-H185, Olympus Corporation). Before the procedure, all patients received prophylactic antibiotic treatment (ciprofloxacin or ceftriaxone). Samples of the material contained in the PPFC were collected for microbiological, cytological, and laboratory analyses.

### 2.5. Transmural Drainage with the Single Transluminal Gateway Technique (SGT) [7, 26]

**Figure 7:**
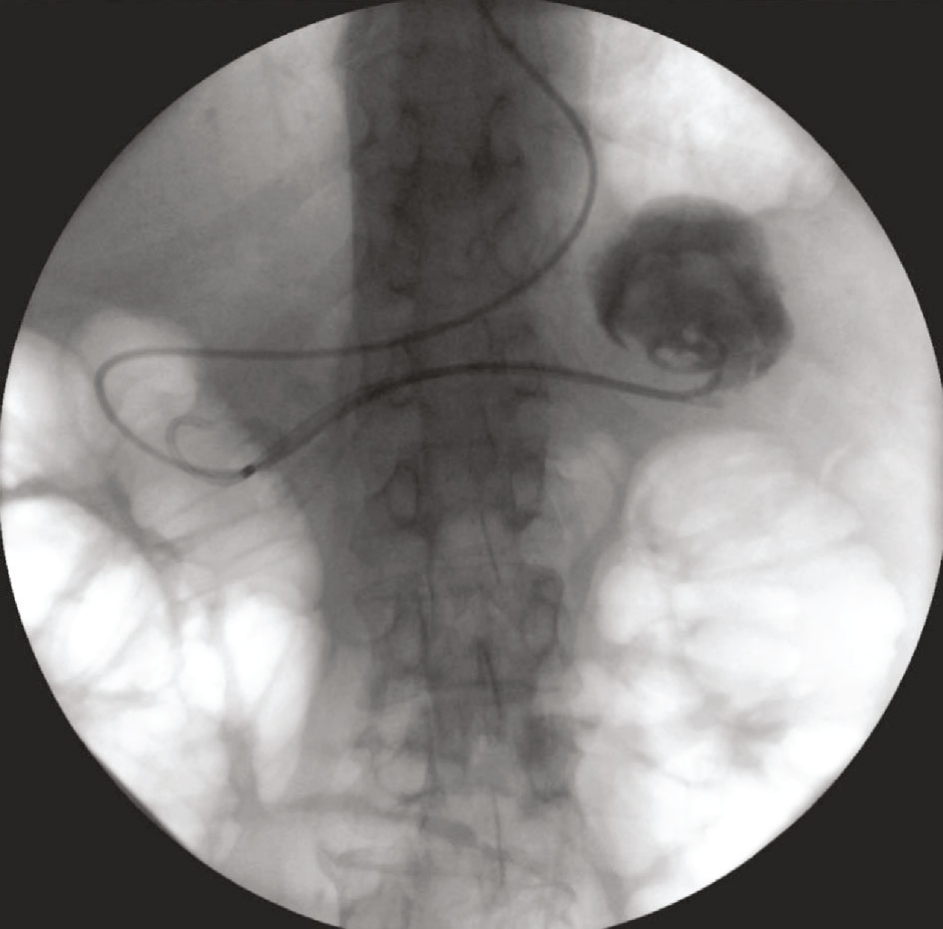
Active transpapillary drainage of a pseudocyst located in the pancreatic tail.

**Figure 8:**
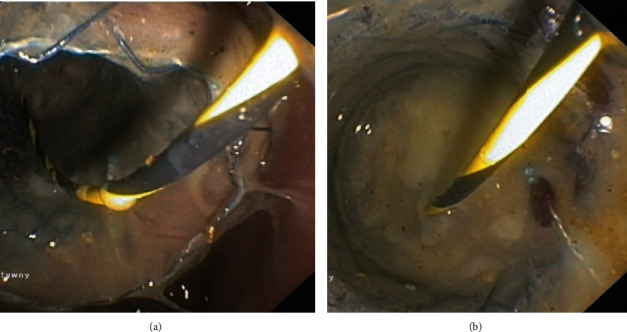
(a, b) Single transluminal gateway technique using LAMS for infected pancreatic pseudocyst treatment.

Placement of the pancreaticogastric or pancreaticoduodenal anastomosis in the form of transmural cystostomy was performed under EUS guidance. The anastomosis between the gastrointestinal lumen and the collection cavity was created with a 10 Fr cystotome (Cystotome CST-10, Cook Endoscopy Inc., North Carolina, USA) and then dilated with a high-pressure balloon with a diameter of up to 15 mm (Cook Endoscopy or Boston Scientific). Through the stomy, a transmural metal endoprosthesis (LAMS) was inserted, measuring 16 mm in diameter and 20 mm, 30 mm, or 40 mm in length (Taewoong Medical or Olympus) (Figures 7 and 8). For active transmural drainage, a 7 Fr or 8.5 Fr nasal drain (Cook Endoscopy) and 7 Fr or 8 Fr double-pigtail stents (Cook Endoscopy) were inserted into the collection cavity through the LAMS. In the case of passive transmural drainage, only 7 Fr or 8.5 Fr double-pigtail stents (Cook Endoscopy) were used through LAMS.

### 2.6. Multiple Transluminal Gateway Technique (MTGT) [7, 26–29]

In patients with additional transmural stomy created between the collection and lumen of the gastrointestinal tract, the placement of the anastomosis was also decided under EUS guidance. The transmural cystostomy was created with a 10 Fr cystostome (Cystotome CST-10, Cook Endoscopy) and expanded with a high-pressure balloon with a diameter of up to 15 mm (Boston Scientific, Massachusetts, USA). Next, a metal endoprosthesis (LAMS) with a diameter of 16 mm and length of 30 mm or 40 mm (Taewoong Medical or Olympus) was inserted transmurally. Depending on the type of drainage, a 7 Fr or 8.5 Fr nasal (Wilson-Cook) and/or 7 Fr or 8 Fr double-pigtail stent (Wilson Cook) drain was inserted through the endoprosthesis and into the collection lumen.

### 2.7. Direct Endoscopic Necrosectomy (DEN) [7, 26, 30–33]

Direct endoscopic necrosectomy procedures, which mechanically remove necrotic tissue, were performed in WOPN patients with no clinical improvement despite the drainage treatment or even if the necrotic collections became infected. The first stage of DEN involved removing the nasal drain. Through the transmural stomy with the LAMS inside, a gastroscope was introduced into the necrotic area. The necrotic collection cavity was subsequently flushed multiple times with saline solution, and the washings were removed by suction. A 15–20 mm extraction balloon (Cook Endoscopy) and Dormia basket (Cook Endoscopy or Olympus) were used to remove necrotic tissue under direct endoscopic image guidance. This procedure was repeated several times. Upon completion, the nasal drain and/or double pigtail plastic stents were reinserted transmurally.

### 2.8. Drainage System

When active transmural drainage was used, the PPFC was flushed with saline (60–200 mL) through the nasal drain every 2 hours during the first 48 hours of the postoperative period and every 4 to 6 hours on the following days. If the patient's clinical symptoms suggested PPFC infection, the antibiotic therapy was prolonged or the contents of the collection were cultured again with antibiotic susceptibility testing.

### 2.9. Treatment Efficacy Assessment

During active transmural drainage, the size of the fluid collection was measured every seven days via abdominal ultrasound. Abdominal CECT was used to confirm complete regression of the fluid collection or in cases where the patient's clinical condition deteriorated despite ongoing treatment. Active drainage was discontinued once clinical success could be established, while the patients were still on passive transmural drainage. After four weeks, an endoscopic procedure was performed during subsequent hospitalization and the passive transmural drainage was either continued (with transmural endoprostheses replaced) or discontinued (with the transmural endoprostheses removed). The decision to continue passive transmural drainage was made depending on the fluid collection size and the presence of any disruption in the MPD, as revealed during ERP. If the PPFC persisted in residual form (–30–40 mm) or recurred (>40 mm), passive endoscopic drainage was continued and the transmural endoprostheses were replaced for another four weeks. In cases of complete PPFC regression, an endoscopic procedure was performed to remove the transmural endoprostheses and passive endoscopic drainage was completed.

### 2.10. Definitions

Technical success was defined as successful placement under endoscopic and radiologic image guidance of the transmural stent with its distal and proximal ends located in the PPFC cavity and lumen of the gastrointestinal tract (stomach or duodenum), respectively. A procedure was confirmed to be technically successful if the contrast agent administered was flowing freely from the PPFC through the transmural stent without leaking out of the gastrointestinal tract or the stent.

Clinical success was defined as resolution of complaints associated with the presence of the PPFC and complete regression of the collection or its diameter decreasing to <40 mm in imaging tests.

Long-term success was defined as the absence of complaints and complete PPFC regression or its size decreasing to <40 mm during follow-up after the end of the endoscopic drainage.

Recurrence of fluid collection was understood as a collection size of >40 mm or reappearance of symptoms during follow-up.

Transmural stent dislocation was defined as the spontaneous migration of the transmural stent away from the anastomosis between the gastrointestinal lumen and PPFC cavity.

Early dislocation of the transmural stent was established if dislocation occurred within the first seven days following the procedure of endoscopic transmural drainage.

Late dislocation of the transmural stent was established if dislocation occurred more than seven days after the procedure.

Proximal stent dislocation was defined as migration of the transmural endoprosthesis from the anastomosis into PPFC lumen, where both flanges of the stent were inside the collection cavity and away from the gastrointestinal wall.

Distal stent dislocation was defined as migration of the transmural endoprosthesis from the anastomosis into the lumen of the gastrointestinal tract, where both flanges of the stent were inside the gastrointestinal lumen.

### 2.11. Statistical Analysis

All statistical calculations were conducted using the statistical software TIBCO Software Inc. (2017). Statistica software (data analysis software system), version 13, was also used (http://statistica.io). Quantitative variables are characterized using arithmetic means, standard deviation, median, and minimum and maximum values (range). Qualitative variables are presented as numbers and percentages.

## 3. Results

### 3.1. Patient Characteristics

The study enrolled 257 patients with symptomatic postinflammatory PPFCs who underwent an endoscopic transmural drainage procedure performed using LAMS; 188 patients (73.15%; 39 women and 149 men; mean age, 62.02 (21–83) years) were diagnosed with WOPN and 69 (26.85%; 13 women and 56 men; mean age, 60.93 (20–78) years) with pancreatic pseudocysts. The mean time from the onset of pancreatitis to the start of endotherapy (ET) was 76 (29–411) days. Chronic pancreatitis was diagnosed in 72 patients (28.02%). Detailed patient characteristics are presented in Table 1.

An infection diagnosed on the basis of a positive culture of the PPFC contents was present in 115 patients with WOPN and in 42 patients with pancreatic pseudocysts. In both groups, the most common bacterial pathogens isolated from the fluid sample were *Escherichia coli*, *Klebsiella pneumoniae*, *Enterococcus faecalis*, and *Staphylococcus epidermidis*. The remaining indications for endoscopic treatment are shown in Table 2. A total of 112 patients (43.58%) presented with more than one indication for ET.

### 3.2. Endoscopic Treatment Technique

All 257 patients underwent endoscopic transmural drainage of postinflammatory PPFCs (transgastric in 223 patients and transduodenal in 34 patients). Twenty-seven patients with sterile pancreatic pseudocysts underwent passive transmural drainage initially. Active transmural drainage was performed in 230 patients (188 patients with WOPN and 42 patients with infected pancreatic pseudocysts). Additional active transpapillary drainage was performed in 11 patients (Figure 7) and an additional percutaneous drainage in 24 patients, and all 230 patients continued passive transmural drainage discontinuing active drainage.

**Figure 9:**
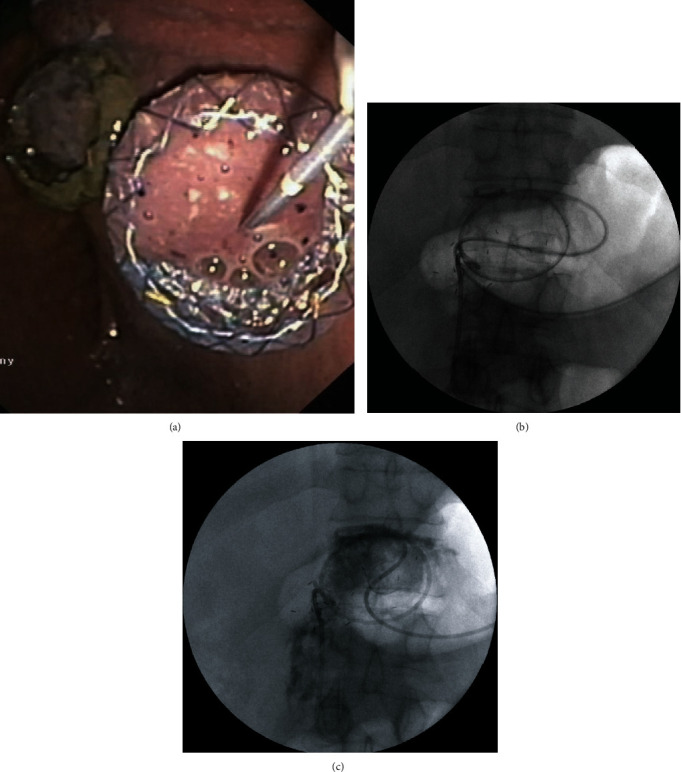
(a–c) MTGT using two LAMS in infected WOPN treatment.

**Figure 10:**
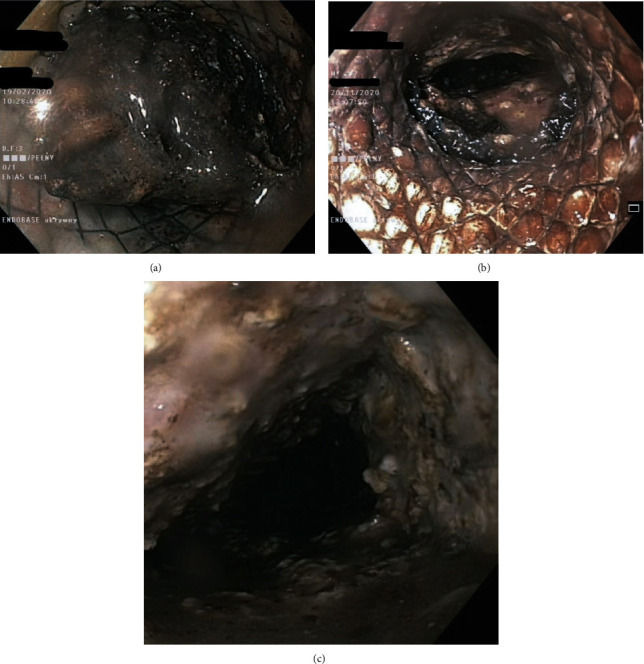
(a–c) DEN of an infected pancreatic necrosis. (a, b) Endoscopic image after insertion of a gastroscope into the lumen of the LAMS. (c) Image from the lumen of the infected WOPN after the gastroscope is delivered through the stent into the fluid collection.

Single transluminal gateway techniques (Figures 8(a) and 8(b)) were applied to 167 patients. Multiple transluminal gateway techniques (Figures 9(a)–9(c)) were used in 90 patients. DEN (Figures 10(a)–10(c)) was performed in 103 patients with WOPN.

### 3.3. Duration of Endotherapy

Active endoscopic drainage took an average of 13.34 (5–82) days. The average duration of passive transmural drainage was 84 (25–281) days. The mean number of endoscopic procedures was 8.61 (2–28). During the endoscopic treatment of the 257 patients with PPFCs, 942 LAMS were used.

### 3.4. Endoscopic Treatment Complications

Complications during endoscopic transmural drainage were observed in 34 patients (13.23%). Of these, a vast majority were stent-related complications, constituting 32 of the complicated cases. Among the 34 patients who experienced endotherapy complications, 8 required surgical treatment. Detailed information on the complications is presented in Table 3.

### 3.5. Gastrointestinal Bleeding

**Figure 11:**
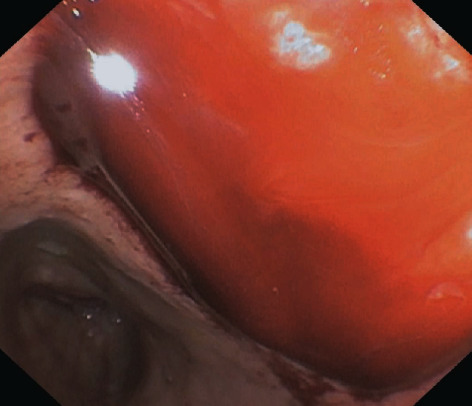
Endoscopic image (gastroscopy). Arterial bleeding from the collection cavity through the transmural LAMS into the gastric lumen.

The most common complication of endoscopic treatment was bleeding into the upper gastrointestinal tract, which was observed in 20 patients. For all cases, the cause was bleeding from the PPFC through transmural cystostomy into the gastrointestinal lumen (Figure 11).

**Figure 12:**
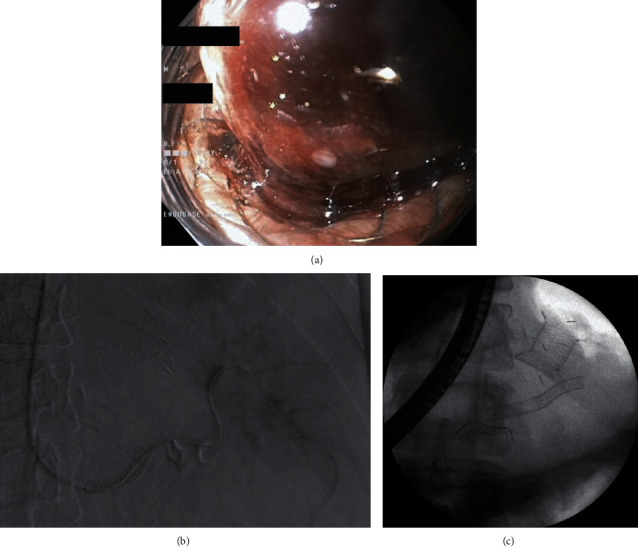
(a–c) Bleeding from the splenic artery during transmural drainage of a pancreatic necrosis. (a) The endoscopic image reveals a blood clot inside of the transmural stent. (b, c) The patient received endovascular treatment by inserting a stent graft to bypass the damaged vessel.

Conservative treatment with blood transfusions and blood derivatives proved successful in 8 patients with gastrointestinal bleeding during ongoing transmural drainage. Endoscopic treatment with hemostatic powder (*Hemospray*, Cook Endoscopy) sprayed into the collection cavity was effective for managing bleeding in 5 patients. Another 5 patients required endovascular treatment with embolization of the perforated vessel (4 cases) or insertion of a stent graft to bypass the site of vascular rupture (1 case) (Figures 12(a)–12(c)). Among the patients who received endovascular treatment, 4 had bleeding from the splenic artery and 1 from the gastroduodenal artery. Due to the inefficacy of minimally invasive bleeding management techniques, 2 patients required surgical treatment. During laparotomy, the bleeding artery (the gastroduodenal artery in 1 case and the splenic artery in 1 case) was ligated using the stick tie technique.

### 3.6. Early Dislocation of the Transmural Stents

**Figure 13:**
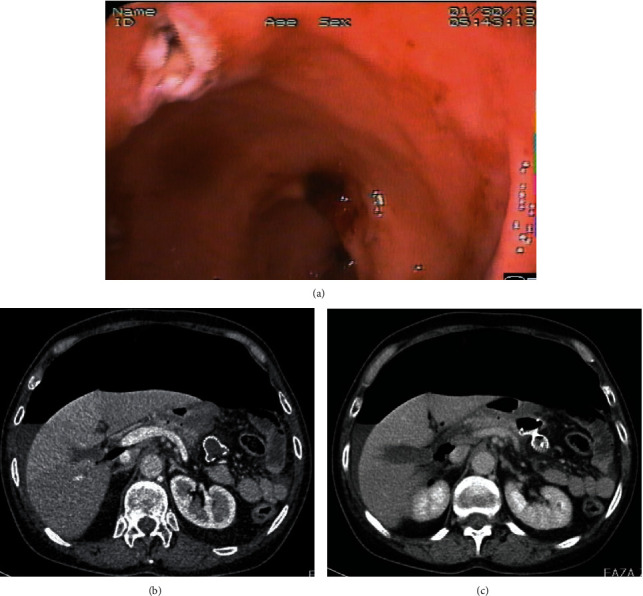
(a–c) Early proximal transmural stent migration. (a) The endoscopic image shows the transmural fistula without the stent. (b, c) Contrast-enhanced multiphase computed tomography image of the abdominal wall in a patient suffering from a perforation of the gastrointestinal tract due to early proximal dislocation of the transmural stent. (b, c) A large amount of air can be seen in the peritoneal cavity, as well as the dislodged transmural stent outside of the gastrointestinal lumen.

**Figure 14:**
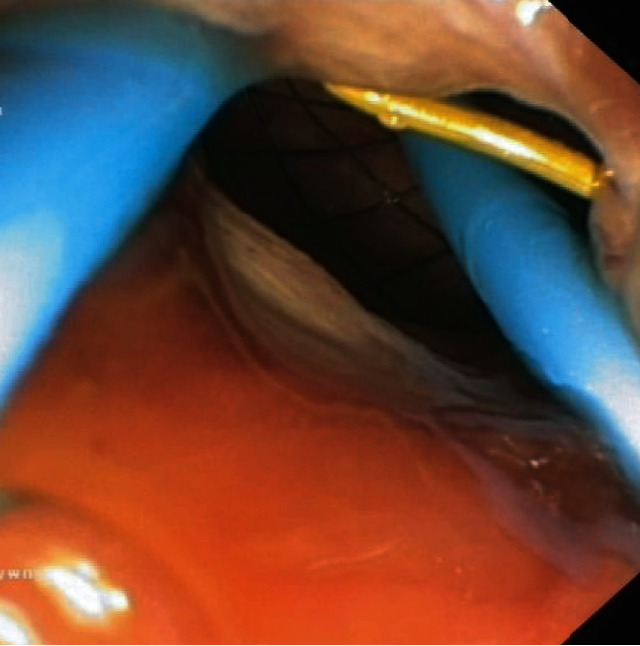
Endoscopic image of early proximal dislocation of the transmural stent into the collection cavity.

Of the 34 patients with complications, 7 developed a perforation of the gastrointestinal tract due to early dislocation of the transmural stent (Figures 13(a)–13(c)). Six patients developed proximal stent dislocation into the lumen of the PPFC (Figure 14). One patient developed distal dislocation of the transmural stent into the lumen of the gastrointestinal tract.

**Figure 15:**
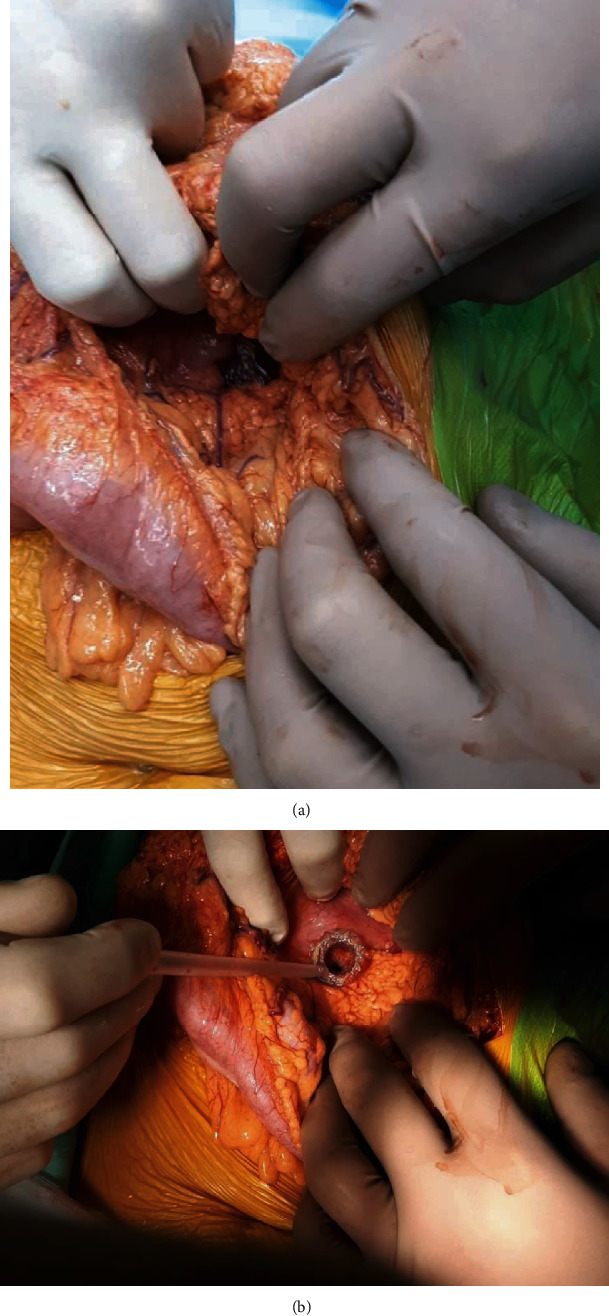
(a, b) Intraoperative image of a laparotomy performed in a patient suffering from early proximal dislocation of the transmural stent. The dislodged stent can be seen.

Endoscopic treatment to remove the dislodged stent and insert a new transmural endoprosthesis, accompanied by percutaneous decompression of the peritoneal cavity, proved to be an effective treatment method in 2 patients. The other 5 patients with early transmural stent dislocation required surgical treatment. All 5 patients had sutured gastrointestinal perforation, and the transmural stent was removed, while external (percutaneous) drainage was used to treat the pancreatic fluid collection. Among the 5 patients who underwent surgical treatment for early transmural stent dislocation, 3 required a laparotomy (Figures 15(a) and 15(b)) and 2 underwent the procedure successfully performed from laparoscopic access.

### 3.7. Pancreatic and Peripancreatic Fluid Collection Perforation

PPFC perforation with fluid leakage from the collection cavity into the retroperitoneal space was found in 2 patients. One of these patients required surgical treatment; laparotomy was performed with drainage and rinsing of the retroperitoneum. The other patient underwent successful percutaneous drainage of the retroperitoneal space without the need to resort to surgical treatment.

### 3.8. Late Dislocation of the Transmural Stents

**Figure 16:**
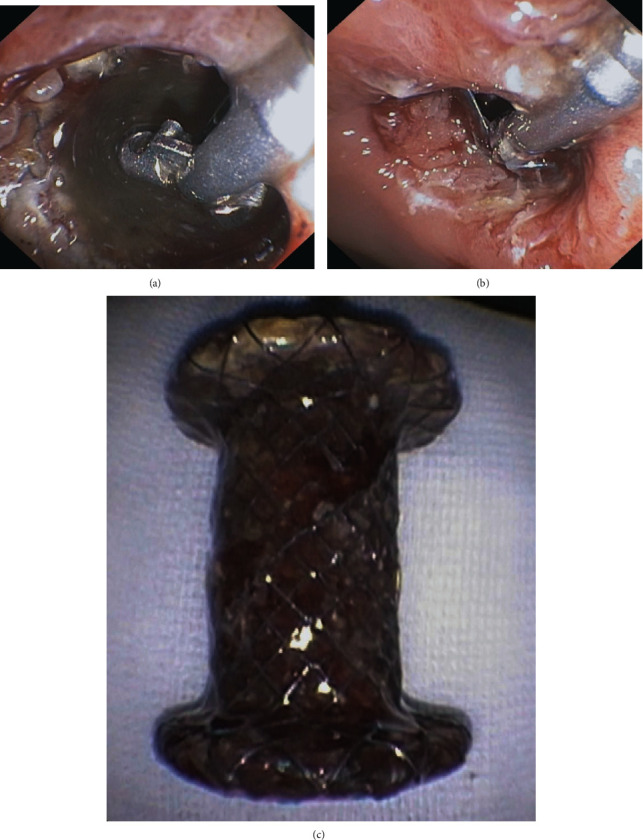
(a–c). Endoscopic treatment of late proximal transmural stent dislocation. (a, b) After the stent was grasped with endoscopic forceps, (c) it could be removed.

Five of the 34 complicated cases developed late dislocation of the transmural stent. Two of these patients were diagnosed with distal dislocation of the stent into the gastrointestinal lumen. The remaining three patients had proximal dislocation of the transmural stent into the collection cavity. The average time from the procedure to the diagnosis of late dislocation was 17 (10–27) days. In all dislocation cases, an endoscopic procedure was performed wherein the dislodged stent was grasped with rat tooth forceps and pulled outside (Figures 16(a)–16(c)).

### 3.9. Efficacy of Endotherapy

Technical success of the transmural drainage procedure was achieved in 255 patients (99.22%). Clinical success was achieved in 242 patients (94.16%).

### 3.10. Mortality

Mortality during ET was observed in 8 patients (3.11%) and was not associated with ongoing endoscopic treatment. All fatal cases reported were caused by multiple organ failure during the course of severe acute necrotizing pancreatitis.

### 3.11. Long-Term Success

During the follow-up period, which lasted an average of 213 (32–1034) days, long-term success of PPFC ET was achieved in 221 patients (85.99%). PPFC recurrence was reported in 17 patients during follow-up. Of these, 15 patients underwent successful endoscopic treatment for recurrent fluid collection. In two patients, the recurrent PPFC necessitated surgical treatment.

## 4. Discussion

The choice of drainage technique in patients with postinflammatory PPFCs should rely primarily on experience of the treating medical center [7–13, 26–33]. This paper shows that ET can be an effective minimally invasive treatment for such patients. Despite the high efficacy of such treatments, its safety offers some significant space for improvement. As discussed above, most complications during endoscopic treatment of PPFCs are associated with the design of the transmural endoprosthesis. Therefore, it is reasonable to pursue further improvements in the quality of endoscopic equipment to minimize the incidence of complications. Meanwhile, efforts to advance the safety of endoscopic treatment with next-generation novel stent designs may contribute to greater efficacy of this treatment.

Traditionally, endoscopic transmural drainage of postinflammatory PPFCs has been performed using plastic (teflon or polyethylene) double-pigtail stents [7, 15–18]. The most commonly used procedure involves transmural insertion of several plastic stents to maintain the patency of the pancreaticogastric or pancreaticoduodenal cystostomy and to ensure undisturbed outflow of the fluid from the collection cavity into the gastrointestinal tract [15–18]. The wider the fistula, the more efficient is the transmural drainage [21, 22].

As a result of advancements in biomedical materials, SEMSs were introduced to the market [19–23, 34–41]. Currently, interventional treatment in gastroenterological endoscopy relies on SEMSs, which come in fully covered, partially covered, and uncovered versions [34, 35]. Uncovered SEMSs offer lower risk of migration, resulting from their higher potential for tissue overgrowth, but often lead to a shorter duration of patency, making it impossible to remove or replace the stent [34, 35]. Fully covered SEMSs are more prone to migration because they are covered with a special polymer coating that prevents tissue overgrowth and prolongs patency, while simultaneously facilitating removal or replacement [34, 35]. A sort of compromise is offered by partially covered SEMSs, which are usually nonremovable but less prone to migration or tissue overgrowth, which ensures longer duration of patency.

With this, only the fully covered type can find application in transmural drainage of postinflammatory PPFCs, where the SEMS must be removed upon treatment completion [7, 19–23]. A special polymer membrane that fully coats the stent not only prevents tissue overgrowth but also ensures leak-proof quality of the connection, precluding any leakage of the collection fluid outside the gastrointestinal tract. SEMSs for transmural drainage are specially designed to ensure maintenance of the large width of the cystostomy [19–23]. Owing to the two-flange design of the transmural LAMS with the proximal flange oriented towards the gastrointestinal lumen and the distal flange into the collection cavity, the distance between the gastrointestinal wall and the wall of the PPFC at the site of the transmural fistula can be kept stable [19–23]. These benefits associated with the use of LAMS offer an advantage over plastic “double-pigtail” stents in terms of endoscopic treatment outcomes in the management of PPFCs [19–23]. The use of fully covered SEMS (LAMS) versus traditional plastic endoprostheses in transmural drainage improves treatment results in patients with postinflammatory PPFCs, most notably in the course of acute necrotizing pancreatitis [19–23]. Despite the good outcomes of transmural drainage with the use of LAMS, every type of stent has its own strengths and weaknesses and selection of the right endoprosthesis remains a challenge [7, 19–23]. This paper discusses complications observed during endotherapy of postinflammatory PPFCs, which were largely connected with the design of the transmural SEMS (LAMS) applied. Polymers and other biomedical materials are constantly evolving and next-generation endoprostheses may contribute to improvements in clinical outcomes. It appears that some of the challenges discussed in this publication might be resolved owing to new technologies being developed and implemented in the field of biomedical materials.

The two most common and most serious groups of complications associated with the endoscopic treatment of PPFCs are gastrointestinal bleeding [42] and perforations caused by leaking pancreaticogastric or pancreaticoduodenal anastomoses, which are usually due to dislocation of the transmural stent [19, 20, 23, 36–41].

With regard to gastrointestinal bleeding during transmural drainage of postinflammatory PPFCs, great progress in reducing the incidence of complications has been achieved with the advent of EUS techniques [16, 17]. EUS guidance during procedures of transmural access into PPFCs with Doppler imaging allows for a detailed assessment of blood vessels and blood flows. This makes it possible to circumvent these structures when creating the anastomosis [16, 17]. Using EUS guidance during transmural drainage of PPFCs limits the incidence of treatment complications, particularly hemorrhages associated with vascular perforations that occur while creating transmural cystostomy [17]. Despite the development of advanced endoscopic techniques and devices, the high rates of bleeding into the PPFC lumen remain a major challenge in transmural drainage treatment. This type of complication is often caused by blood vessels adjacent to the fluid collection being damaged by the distal flange of the LAMS. While inserting a plastic double-pigtail stent through the LAMS limits the risk of this kind of complication by moving the back wall of the PPFC away from the distal flange, PPFC cavity bleeding during transmural drainage is still a major complication associated with a high risk of fatal outcomes. However, small blood vessels are commonly damaged and most bleeding complications of endoscopic drainage can be treated with conservative methods. If this strategy is ineffective and if the bleeding originates from a small vessel or granulation tissue of the healing collection wall, endoscopic treatment usually yields good results. In hemodynamically unstable patients with massive bleeding from the large arteries into the pancreatic collection cavity, interventional treatment is necessary. The method of choice in these circumstances is endovascular treatment or surgery.

The application of new polymers in manufacturing transmural stents in the form of additional layers of coating to the distal flange of the LAMS will most certainly limit the risk of PPFC cavity bleeding during transmural drainage procedures. This will make it less likely for the distal flange of the LAMS to injure the back wall of the PPFC, and it will no longer be necessary to insert a plastic double-pigtail stent through the lumen of the LAMS. The thick polymer coating of the most protruding part of the distal flange will then take over the function hitherto performed by the additional plastic stent, which can lower the costs of endoscopic treatment of PPFCs.

Another major complication of transmural drainage with LAMS is gastrointestinal tract perforation due to transmural stent dislocation that migrates outside of the transmural anastomosis [43–45]. Gastrointestinal perforation is most commonly associated with early dislocation of the transmural stent occurring during the first week following the endoscopic transmural drainage procedure. Late transmural stent dislocation, which occurs more than one week after the procedure, is less likely to result in gastrointestinal perforation. This is because, after a week, the site of anastomosis between the gastrointestinal tract and the PPFC is healed and sufficiently tight to prevent the absence of the stent from causing the gastrointestinal wall to move away from the collection wall and allow air to escape the gastrointestinal lumen.

Treating transmural stent dislocation should primarily depend on the patient's clinical condition. In stable patients with an early transmural stent dislocation and air leaking out of the gastrointestinal lumen, as revealed by imaging, endoscopic treatment can be attempted to adjust the position of the dislodged stent or add another stent using the stent-in-stent technique. If this strategy is successful, it is also necessary to remove air from the peritoneal cavity through a percutaneous incision. Endoscopic treatment of early dislocations of LAMS, despite favorable short-term outcomes, usually prove ineffective on long-term follow-up, thus leaving surgical treatment as the method of choice. This paper describes an effective method of surgical treatment for early dislocation of the LAMS, consisting of suturing the perforated site (transmural cystostomy) within the upper gastrointestinal tract and removing the dislodged transmural stent through laparotomy or, preferably, through laparoscopic access. The surgery also involves percutaneous drainage (external) of the PPFC. Subsequently, in the postoperative period, while the external drainage is still ongoing, an endoscopic procedure is performed, whereby internal drainage (transpapillary or transmural) of the PPFC is provided. Upon internal drainage completion, the external drainage was removed. This is how external drainage is replaced with the PPFC internal drainage.

In cases of late transmural stent dislocation occurring more than a week after the procedure, endotherapy is generally an effective method of treatment. Proximal stent migration occurs when the transmural stent migrates into the PPFC lumen. Endoscopic treatment of late proximal dislocation of the transmural stent involves inserting another transmural stent through the transmural cystostomy or creating another cystostomy between the gastrointestinal lumen and the PPFC cavity. Through the transmural endoprosthesis, an endoscope is inserted into the PPFC under the guidance of endoscopic imaging. Different types of endoscopic tools are used to capture and remove the dislodged stent. In cases of late distal dislocation of the transmural LAMS where the stent migrates into the gastrointestinal lumen and if the dislodged stent is located within the upper gastrointestinal tract, it can still be removed with the use of endoscopic techniques. However, if the dislodged stent has migrated further down the gastrointestinal tract and beyond the duodenojejunal flexure (ligament of Treitz), the patient is usually monitored until the stent is spontaneously passed along the entire gastrointestinal tract without any complications. If these strategies fail, surgical treatment remains the method of choice.

As one follows the continuous dynamic development of biomedical technologies, it can be presumed that as the design of transmural stents evolves towards a larger diameter and size of both flanges and their improved shape, it will become possible to limit the risk of early and late dislocations of transmural LAMS in terms of both distal and proximal migrations. Moreover, a sufficiently larger lumen diameter of the LAMS prevents it from being obstructed by necrotic tissues.

According to the guidelines for endotherapy of postinflammatory PPFCs, transmural LAMS should be either replaced or removed after 8–12 weeks [13, 31, 36–41]. This paper describes a management technique in which the LAMS used for transmural drainage was replaced or removed every four weeks, which lowered the risk of endotherapy complications. The frequent replacement or removal of transmural stents prevents the so-called buried LAMS syndrome, which is a condition caused by the stent becoming overgrown with tissue despite its layer of coating [31, 36–41].

This paper addresses the challenges and issues faced by endoscopists performing transmural drainage using LAMS. From the endoscopist's perspective, a major challenge in transmural drainage of PPFCs is often the right placement of the stent. This is a crucial stage in determining the technical success of the procedure. Once the transmural cystostomy is performed and the guidewire is inserted into the PPFC, the transmural stent is introduced. Considering the challenging anatomical conditions and rigid nature of the stent delivery system used to introduce the unexpanded LAMS, this stage requires particular caution while expanding the stent. Failure of any kind has the potential to cause early dislocation of the stent during the endoscopic procedure, which can lead to gastrointestinal perforation. At this point, this depends on the experience and skill of the endoscopist. While the construction and technical features of the stent delivery system can be expected to improve, the difficult anatomical environment will not. In particular, PPFCs are located away from the gastrointestinal wall, which do not form a discernible bulge on the gastric or duodenal wall and are situated within the distal part of the pancreatic body or within the tail, where transmural access can usually be obtained from the subcardiac region of the stomach, often with endoscopic inversion. A major convenience with regard to improving the quality and safety of endoscopic transmural drainage with LAMS is the controlled release system used in certain types of SEMS, which allows for accurate and controlled placement of the transmural stent in the desired location. This solution limits the risk of transmural stent dislocation and consequently reduces the potential for gastrointestinal perforations and leaks within the pancreaticogastric or pancreaticoduodenal anastomosis. Despite these benefits, controlled release systems have not yet been featured in LAMS for transmural drainage.

## 5. Conclusions

This paper discussed the high efficacy of ET and its potential complications, which are mostly related to the design of the LAMS used. The high efficacy of LAMS in the transmural drainage of PPFCs is associated with lower treatment safety. Most ET complications respond to conservative or minimally invasive treatments, including endoscopic techniques. Surgical treatment of this type of complication remains the method of choice if other treatment options fail.

This paper discusses possible directions of development in the field of transmural LAMSs. It may also be helpful in addressing the possible expectations of the interventional endoscopist towards stent designers and manufacturers. High hopes for improving the quality of endoscopic equipment are placed in the development of new technologies from biomedical materials, including polymers, equipment for the production of equipment. Possibly, subsequent novel endoprosthesis projects, based on the above results, will be able to meet the current needs and requirements associated with endoscopic endoscopic transmural drainage procedures in cases of postinflammatory PPFCs. The ultimate goal is to improve the safety of minimally invasive techniques for the treatment of the local consequences of pancreatitis. Hopefully, our findings will contribute to development of novel and original transmural LAMS designs.

## Figures and Tables

**Table 1:** Characteristics of the patients with PPFCs.

	All patients (*n* = 257)
Age, mean (range)	61.88 (20–83)
Sex, *n*, men (%)	205 (79.77%)
Etiology, *n*, (%)	
Alcoholic	166 (64.59%)
Nonalcoholic	91 (35.41%)
PPFC size (cm), mean (range)	14.96 (6.4–36.32)
Type of PPFCs	
Pancreatic pseudocyst	69 (26.85%)
Walled-off pancreatic necrosis	188 (73.15%)
Time from the pancreatitis to endotherapy (days), mean (range)	76 (29–411)

**Table 2:** Indications for endoscopic treatment of PPFCs.

Indication	Number of patients, *n* (%)
Infection	157 (61.09%)
Subileus/ileus	84 (32.68%)
Icterus	21 (8.17%)
Abdominal pain	121 (47.08%)
Weight loss	101 (39.3%)
Other	9 (3.5%)

**Table 3:** Complications of endoscopic treatment of patients with pancreatic fluid collections.

Complication	Number of patients	Treatment	Number of patients
Upper gastrointestinal bleeding	20	Conservative	8
		Endotherapy	5
		Endovascular treatment	5
		Surgical	2
Early dislocation of LAMS	7	Endotherapy	2
		Surgical	5
Perforation of PPFC	2	Percutaneous drainage	1
		Surgical	1
Late dislocation of LAMS	5	Endotherapy	5

## Data Availability

Data are available on request (corresponding author email: matjagiel@gmail.com).
